# Green Smoke, Red Flag: Cannabis and the Risk of Orbitopathy and Dermopathy in Thyrotoxicosis

**DOI:** 10.7759/cureus.39092

**Published:** 2023-05-16

**Authors:** Victory Okpujie, Sara Ozumba, Oluwatobi A Olaomi, Fidelis E Uwumiro, Adetayo E Ajiboye, Olawale Abesin, Rebecca O Solomon, Olamide M Ogunfuwa, Judith H Hassan, Mojeed R Opeyemi

**Affiliations:** 1 Internal Medicine, Central Hospital Benin, Benin City, NGA; 2 Family Medicine, University of Nigeria, Nsukka, NGA; 3 Radiology, University of Ibadan, Ibadan, NGA; 4 Surgery, Our Lady of Apostles Hospital, Akwanga, NGA; 5 Surgery, College of Medical Sciences, University of Jos, Jos, NGA; 6 Dermatology, Royal Free London NHS Foundation Trust, London, GBR; 7 Internal Medicine, Royal Cornwall Hospitals NHS Trust, Cornwall, GBR; 8 Paediatrics and Child Health, Community Medicine, Lagos State Primary Health Care, Ijora Primary Health Centre, Lagos, NGA; 9 Internal Medicine, Federal Medical Centre Abeokuta, Abeokuta, NGA; 10 Health Sciences and Social Work, Western Illinois University, Macomb, USA

**Keywords:** nationwide inpatient sample, thyroid dermopathy, thyroid orbitopathy, thyrotoxicosis, cannabis use, medical marijuana

## Abstract

Background

The use of cannabis has been associated with an array of multi-systemic physiological effects. However, the medical literature on the potential role of cannabinoids in the management and outcomes of thyrotoxicosis remains scarce. We studied the association between cannabis use and orbitopathy, dermopathy, and the length of hospital stay for thyrotoxicosis admissions.

Methods

A thorough analysis was conducted on adult hospitalizations in 2020 with a primary discharge diagnosis of thyrotoxicosis, using data from the Nationwide Inpatient Sample (NIS). To ensure data completeness and consistency, hospitalizations with missing or incomplete information, as well as those involving patients under 18 years of age, were excluded from the study. The remaining study sample was categorized into two groups based on the presence or absence of cannabis use, as determined by ICD-10-CM/PCS codes. Subtypes of orbitopathy, dermopathy, and potential confounding factors were identified based on previous literature and defined using validated ICD-10-CM/PCS codes. The association between cannabis use and the outcomes was evaluated using multivariate regression analysis. The primary focus was on thyroid orbitopathy, while dermopathy and the average length of hospital stay were considered as secondary outcomes.

Results

A total of 7,210 hospitalizations for thyrotoxicosis were included in the analysis. Among them, 404 (5.6%) were associated with cannabis use, while 6,806 (94.4%) were non-users serving as controls. Cannabis users were predominantly female (227, 56.3%), which was similar to the control group (5,263, 73%), and they were primarily of Black descent. Notably, the cohort of cannabis users was significantly younger than the control group (37.7 ± 1.3 vs. 63.6 ± 0.3). Upon conducting multivariate regression analysis, it was found that cannabis use was linked to a significant increase in the odds of orbitopathy among patients with thyrotoxicosis (AOR: 2.36; 95% CI: 1.12-4.94; P = 0.02). Additionally, a history of tobacco smoking was also correlated with higher odds of orbitopathy in the study (AOR: 1.21; 95% CI: 0.76-1.93; p = 0.04). However, no significant association was observed between cannabis use and the odds of dermopathy (AOR: 0.88; 95% CI: 0.51-1.54; p = 0.65) or the average length of hospital stay (IRR: 0.44; 95% CI: 0.58-1.46; p = 0.40).

Conclusion

The study identified a significant association between cannabis use and increased odds of orbitopathy in patients with thyrotoxicosis. Additionally, a history of tobacco smoking was also found to be correlated with augmented odds of orbitopathy.

## Introduction

Cannabis is a psychoactive substance that is used extensively for both medicinal and recreational purposes, either in its natural form, commonly referred to as marijuana, weed, pot, or ganja, or its synthetic forms, such as Spice, K2, or Kronic [1]. Cannabis use has been found to have various physiological effects on the human body, including the eyes, skin, and soft tissues [2]. Researchers have identified two G protein-coupled cannabinoid receptors, cannabinoid receptor 1 (CB1) and cannabinoid receptor 2 (CB2), which are widely distributed in mammalian tissue [3,4]. According to several published scientific sources, cannabis use can cause changes in the eyes, such as redness, dryness, decreased corneal endothelial density, motility deficits, decrements in smooth pursuit and saccadic eye movements, and decreased intraocular pressure (for which researchers have explored a potential benefit in glaucoma treatment) [5-7].

The existing body of research investigating the effect of cannabis use on thyroid function has produced conflicting findings, leading to a lack of consensus on the topic. Some studies have indicated a potential association between cannabis use and alterations in thyroid hormone levels [8,9]. Specifically, elevated levels of thyroid-stimulating hormone (TSH) and decreased levels of free thyroxine (FT4) have been reported. These findings suggest possible disruptions in the hypothalamic-pituitary-thyroid (HPT) axis. However, the existing studies have failed to observe significant associations between cannabis use and thyroid dysfunction [10]. Moreso, the available studies are limited by small sample sizes, heterogeneous populations, and variations in study designs and methodologies. As such, further well-designed and comprehensive research is warranted to elucidate the potential impact of cannabis use on thyroid function, taking into account potential confounding factors and employing standardized measurement protocols.

One of the distressing manifestations of thyrotoxicosis is orbitopathy, also known as Graves' orbitopathy or thyroid-associated ophthalmopathy (TAO), which can significantly impact the quality of life of affected individuals. The pathogenesis of orbitopathy involves autoimmune mechanisms, modulation of fibroblasts, and fibrosis of the levator muscles in the eye socket. In addition to the autoimmunity and fibroblast modulation mechanisms, thyrotoxicosis-induced levator muscle fibrosis may also contribute to the development of eyelid retraction, lagophthalmos, and exposure keratitis. Similarly, thyrotoxicosis can also cause dermopathy or pretibial myxedema, which is characterized by localized skin thickening, erythema, and induration, most commonly affecting the shins. The pathogenesis of dermopathy is thought to involve autoimmunity and fibroblast modulation, similar to orbitopathy.

There is currently limited research available on the effects of cannabis use in individuals with thyrotoxicosis. We aim to identify the association between cannabis use and the likelihood of orbitopathy, dermopathy, and hospital length of stay for thyrotoxicosis hospitalizations.

## Materials and methods

Study design and data source

This retrospective cohort study aimed to examine the relationship between cannabis use and the odds of developing thyroid-associated orbitopathy (TAO), rates of thyroid dermopathy, and mean length of hospitalization (LOS) in adult patients with thyrotoxicosis in the US. The study utilized the 2020 NIS database, which is developed by the Healthcare Cost and Utilization Project (HCUP), a Federal-State-Industry partnership sponsored by the Agency for Healthcare Research and Quality. The NIS database contains billing data submitted by individual hospitals across the US to statewide data organizations and approximates a 20% stratified sample of discharges from US community hospitals, excluding rehabilitation centers and long-term acute care hospitals [11]. The dataset is further weighted to obtain national estimates [12]. The data for this study was extracted from the 2020 NIS database using the Stata® version 17 software (StataCorp, College Station, TX, USA) and coded using the International Classification of Diseases, 10th Revision, Clinical Modification/Procedure Coding System (ICD-10-CM/PCS).

Study population

The study population included all adult hospitalizations with a primary discharge diagnosis of thyrotoxicosis in the year 2020, excluding hospitalizations for individuals below 18 years of age. This cohort was further divided into two groups based on the presence or absence of a secondary diagnosis of cannabis use, identified using the ICD-10-CM codes.

Outcome measures

The primary outcome measure was the effect of cannabis use on the odds of thyroid orbitopathy. This was defined as the presence of ICD-10 codes for one or more of the following: squint (heterotopia), restrictive strabismus, exophthalmos, orbital congestion, or congestive oculopathy (chemosis, conjunctivitis, periorbital edema, corneal ulcers, optic nerve atrophy, and optic neuritis), myopathy of extraocular muscles, decreased visual acuity (due to dysthyroid optic neuropathy), photophobia, visual loss, color blindness, diplopia, lagophthalmos, ocular pain, and optic nerve compression. Secondary outcome measures included rates and odds of thyroid dermopathy (pretibial myxedema) as well as mean length of hospitalization (LOS).

Statistical analysis

Data analysis was conducted using Stata® version 17 software (StataCorp, College Station, TX, USA), utilizing weighted samples for national estimates and in compliance with HCUP regulations. Comorbidities were calculated as proportions, and the chi-square test was used to compare characteristics between the two groups. Multivariate regression analysis was performed to adjust for possible confounders while analyzing primary and secondary outcomes. The confounders were obtained after a thorough literature review and an initial univariate screen. The confounders included young age (40 years and below), high cholesterol, history of other autoimmune diseases, history of tobacco smoking, insurance status, gut colonization by atypical microbiota (such as *Yersinia enterocolitica* and *Escherichia coli*), other thyroid-associated autoimmunopathy, and the Charlson Comorbidity Index (CCI). A negative binomial regression model was used to adjust for LOS, expressed as an incidence rate ratio (IRR). A threshold of 0.05 was maintained for the statistical significance of the outcomes.

Ethical considerations

This study was conducted using de-identified data from the NIS database, which complies with the Health Insurance Portability and Accountability Act (HIPAA) of 1996 and lacks patient-specific and hospital-level identifiers. As a limited dataset, NIS does not require review by an institutional review board. Therefore, this study was exempt from Institutional Review Board approval.

Data availability statement

We used the 2020 NIS database for this study. The NIS is the largest publicly available, all-payer inpatient care database, containing data on more than six million US hospital stays in 2020. NIS data is available online through the HCUP central distributor at http://www.hcup-us.ahrq.gov.

## Results

Clinical and demographic characteristics of the study population

**Figure 1:**
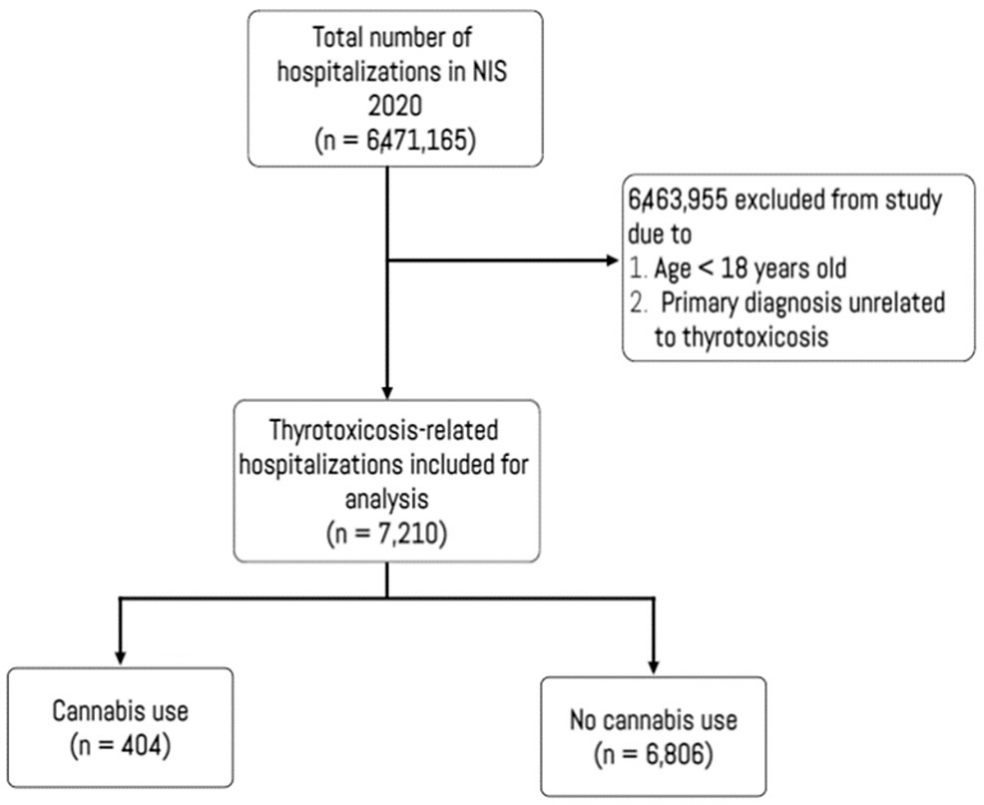
Flowchart depicting inclusion and exclusion criteria for the study NIS, nationwide inpatient sample

This study examined clinical and demographic data from 7,210 thyrotoxicosis admissions, stratified by cannabis use (Figure 1). The prevalence of cannabis use was found to be 5.6% (404), as shown in Table 1.

**Table 1:** Clinical and demographic characteristics of the study cohort All values represent number of hospitalizations (proportion of the subpopulation), except mean age reported in years ± standard deviation ^a^Significant at values <0.05 ^b^*Yersinia enterocolitica* and *Escherichia coli* SD, standard deviation; COPD, Chronic obstructive pulmonary disease $, US dollar

Patient and hospital variables	Total study population (7,210), n (%)	Cannabis use cohort (404), n (%)	P-value^a^
Women	5,263 (73)	227 (56.3)	<0.001
Race/ethnicity			0.03
White	3,129 (43.4)	137 (33.8)	
Black	2,170 (30.1)	189 (46.8)	
Hispanic	1,103 (15.3)	58 (14.3)	
Asian or Pacific Islander	368 (5.1)	0.0 (0.0)	
Native American	79 (1.1)	1.3 (5)	
Other	368 (5.1)	16 (3.9)	
Mean age, (years ± SD)	63.6 ± 0.3	37.7 ± 1.3	0.02
Young age (< 40 years)	2,610 (36.2)	252 (62.5%)	< 0.001
Charlson comorbidity index score			0.22
0	3,468 (48.1)	207 (51.3)	
1	1,989 (27.6)	137 (33.8)	
2	807 (11.2)	25 (6.3)	
≥ 3	944 (13.1)	36 (8.8)	
Comorbidities			
Acute myocardial infarction	404 (5.6)	15 (3.8)	0.45
Congestive heart failure	1,499 (20.8)	96 (23.8)	0.49
Peripheral vascular disease	238 (3.3)	5 (1.3)	0.29
Cerebrovascular disease	187 (2.6)	5 (1.3)	0.43
Dementia	209 (2.9)	0 (0)	0.10
COPD	1,182 (16.4)	40 (10)	0.11
Rheumatoid disease	144 (2)	0 (0)	0.18
Peptic Ulcer Disease	36 (0.5)	5 (1.3)	0.31
Chronic Liver Disease	231 (3.2)	11 (2.6)	0.44
Uncomplicated Diabetes	843 (11.7)	46 (11.3)	0.91
Complicated Diabetes	339 (4.7)	0 (0)	0.04
Hemiplegia or paraplegia	50 (0.7)	5 (1.3)	0.54
Chronic renal disease	497 (6.9)	25 (6.3)	0.80
Cancer	260 (3.6)	0 (0)	0.14
AIDS	10 (0.1)	5 (1.3)	0.01
Median annual income in patient’s zip code			<0.001
$1 – $45,999	2,386 (33.1)	186 (46.3)	
$46,000 – $58,999	1,816 (25.2)	126 (31.3)	
$59,000 – $78,999	1,701 (23.6)	80 (20)	
≥ $79,000	1,305 (18.1)	12 (3)	
Insurance type			0.01
Medicare	1,874 (26)	55 (13.5)	
Medicaid	2,213 (30.7)	191 (47.3)	
Private including HMO	2,357 (32.7)	115 (28.4)	
Uninsured	771 (10.7)	44 (10.8)	
Hospital region			0.26
Northeast	1,607 (22.3)	61 (15)	
Midwest	1,254 (17.4)	91 (22.5)	
South	2,920 (40.5)	182 (45)	
West	1,427 (19.8)	80 (19.8)	
Hospital bed size			0.004
Small	1,392 (19.3)	96 (23.8)	
Medium	1,932 (26.8)	131 (32.5)	
Large	3,886 (53.9)	177 (43.8)	
Weekend admission	1,506 (20.9)	131 (32.5)	0.01
Risk Factors for Orbitopathy			
History of tobacco use	2,581 (35.8)	253 (62.5)	< 0.001
Family history of thyrotoxicosis	216 (3)	15 (3.8)	0.68
Hypercholesterolemia	1,730 (24)	46 (11.3)	0.01
Other autoimmune diseases	224 (3.1)	15 (3.8)	0.71
Gut colonization by atypical microbes^b^	101 (1.4)	5 (1.3)	0.96
Hospital Location/Teaching Status			0.13
Rural	382 (5.3)	10 (2.5)	
Urban non-teaching	944 (13.1)	30 (7.5)	
Urban teaching	5,883 (81.6)	364 (90)	

The total study population had a mean age of 63.6 years (SD = 0.3), with 73% females and a racial distribution of 56.6% non-White and 43.4% White American descent. A majority of the participants (81.6%) were admitted to urban teaching hospitals, had a CCI of 1 or greater (51.9%), and had at least one risk factor for orbitopathy or dermopathy (67.3%). In contrast, the cannabis use cohort had a significantly younger mean age of 37.7 ± 1.3 years, with 62.5% of individuals being under the age of 40 years, and was predominantly black or Hispanic (61.1%). A majority of the cannabis use cohort (85.1%) had a CCI of 1 or lower, and 82.7% had at least one known risk factor for orbitopathy or dermopathy in comparison to the total study population.

Primary outcome

In the total study population, the prevalence of TAO was 5.6%, while the proportion was higher among the cannabis use cohort, which had a prevalence of 12.5%. The unadjusted odds ratio (OR) of TAO was found to be 2.60 (95% CI: 1.29-5.33; p = 0.01) in the cannabis use cohort. After adjusting for other variables, it was found that cannabis use was independently associated with over a 100% increase in the odds of orbitopathy in patients with thyrotoxicosis (AOR: 2.36; 95% CI: 1.12-4.94; P = 0.02), as shown in Table 2. Furthermore, a history of tobacco smoking was also found to be correlated with increased odds of orbitopathy in the study (AOR: 1.21; 95% CI: 0.76-1.93; p = 0.04).

**Table 2:** Risk-adjusted multivariable regression model for primary and secondary outcomes ^a^Adjusted odds ratio ^b^Significant at values <0.05

Variables	AOR^a^	Standard Error	P-value^b^	95% Confidence Interval
Thyroid-associated Orbitopathy				
Cannabis use	2.36	0.89	0.02	1.12 – 4.94
Age < 40 years	1.32	0.32	0.25	0.82 – 2.13
Family history of Graves disease	0.40	0.41	0.37	0.05 – 2.96
History of tobacco smoking	1.21	0.29	0.04	0.76 – 1.93
Thyroid dermopathy				
Cannabis use	0.88	0.25	0.65	0.51 – 1.54
Age < 40 years	0.91	0.21	0.68	0.59 – 1.42
Hypercholesterolemia	0.80	0.14	0.19	0.57 – 1.12
Gut colonization by atypical microbiota	0.84	0.61	0.81	0.20 – 3.48
Black race	1.42	0.22	0.02	1.05 – 1.93
Asian or Pacific Islander descent	0.50	0.17	0.04	0.26 – 0.95
Charlson index	0.90	0.05	0.05	0.81 – 1.00
Existing orbitopathy	3.99	1.12	<0.001	2.25 – 7.09
Insured	1.32	0.32	0.25	0.82 – 2.11
Median annual income ≥ $79,000	1.78	0.35	0.003	1.21 – 2.61
Hospital region in the south	0.52	0.10	< 0.001	0.36 – 0.75

However, neither young age nor a family history of thyrotoxicosis was found to be significantly correlated with the odds of orbitopathy.

Secondary outcomes

In the study overall, there was a 32% prevalence of thyroid dermopathy (pretibial myxedema), with a higher prevalence of 36.3% (147) observed in the cannabis use cohort. The crude odds ratio of dermopathy among cannabis users in the study was 1.21 (95% CI: 0.77-1.94; p = 0.40). However, on multivariate analysis, cannabis use was not associated with a significant difference in the odds of dermopathy (AOR: 0.88; 95% CI: 0.51-1.54; p = 0.65), as shown in Table 2.

Independent predictors of thyroid dermopathy that were identified in the study included being of Black race, having pre-existing thyroid-associated orbitopathy, or having a median annual income of ≥ $79,000 (augmented odds). Admission to hospitals in the southern region and Asian or Pacific Islander descent were associated with lower odds of dermopathy, as shown in Table 2.

The mean LOS in the study population was 3.9 ± 0.1 days, with a slightly shorter mean LOS of 3.70 ± 0.43 days observed in the cohort of cannabis use. Furthermore, hospitalizations lasting longer than five days were more common in the cannabis use cohort (28.8%) than in the total study population (25%). However, adjusted risk analysis (Table 3) did not show a significant association between cannabis use in thyrotoxicosis and changes in mean hospital stay.

**Table 3:** Mean length of stay with cannabis use as independent variable ^a^Length of hospital stay ^b^Adjusted incidence risk ratio ^c^Significant at values <0.05

Adjusted mean LOS^a^	aIRR^b^	Standard Error	p-value^c^	95% Confidence Interval
Cannabis use	0.44	0.52	0.40	-0.58 to 1.46
Age	0.01	0.01	0.32	-0.01 to 0.04
Gut colonization by atypical microbes^d^	2.30	1.30	0.08	1.25 to 4.84
Higher Charlson comorbidity index	0.72	0.10	<0.001	0.52 to 0.91
Other Autoimmune diseases	3.10	1.30	0.02	1.55 to 5.64
High cholesterol	0.06	0.38	0.87	-0.68 to 0.81
Age < 40 years	-0.05	0.39	0.90	-0.81 to 0.72
Admission day is a weekend	-0.21	0.24	0.40	-0.69 to 0.28
Insured	0.48	0.46	0.30	-0.42 to 1.37
Smoking	-0.63	0.26	0.02	-1.14 to 0.12
Any autoimmunopathy	-0.55	0.25	0.03	-1.04 to 0.06

## Discussion

In recent times, there has been an ongoing debate among researchers about the potential role of delta-9-tetrahydrocannabinol (THC), the primary psychoactive component of cannabis, in the treatment of eye disease. However, there has been considerable variation in opinions and trial outcomes, likely attributable to limited scientific evidence on the subject, legislation restricting the use of cannabinoids, adverse effects observed in trials, and the impracticality of prescribing cannabinoids at the necessary doses required to treat eye disease. A recent investigation exploring the impact of smoking cannabis on visual function discovered that smoking cannabis had a significantly negative impact on all the visual parameters examined [13]. On the other hand, there are reports that acute cannabis intoxication exerts positive effects on nighttime vision, with improved adaptation to darkness and scotopic sensitivity [14,15].

The current study indicates that cannabis use may be a possible risk factor or contributor for thyroid-associated orbitopathy (TAO) in patients with thyrotoxicosis, with a higher prevalence of TAO than the total study population. The crude odds ratio of TAO was 2.60 in the cannabis use group, which remained at 2.36 after adjusting for other confounding variables. Previous research has also proposed a connection between cannabis use and orbitopathy. The endocannabinoid system (ECS) is a potential mechanism underlying the link between cannabis use and TAO, as it plays a critical role in regulating the immune system, with dysregulation of the ECS implicated in several autoimmune diseases, including thyroid disease [16]. Delta-9-tetrahydrocannabinol has been shown to activate the ECS by interacting with cannabinoid receptors CB1 and CB2, thereby influencing the immune response. Chronic activation of the ECS due to cannabis use may contribute to the development of thyroid-associated ophthalmopathy (TAO) in individuals with thyrotoxicosis [17]. CB1 is broadly distributed in ocular structures such as the iris, ciliary body muscle, trabecular meshwork, Schlemm's canal, and ciliary pigmented epithelium, indicating that multiple pathways may be involved in the effects of cannabinoids on TAO [16]. Despite the fact that cannabinoids' potential effects have been demonstrated in various clinical studies [18-21], the precise role of these molecules in the orbital dysregulation of thyrotoxicosis remains unclear and necessitates further clinical trials.

Furthermore, the present study discovered that a history of tobacco smoking was linked to an increased risk of orbitopathy. This aligns with prior research that has shown a strong association between smoking and TAO in patients with Graves' disease [22,23]. Smoking has been found to raise the production of thyroid-stimulating hormone (TSH) receptor antibodies, which play an important role in TAO development [24,25]. Additionally, smoking has been found to impact the course of TAO during treatment in a dose-dependent manner, with smokers having a delayed and substantially poorer treatment response.

While more research is needed to fully understand the relationship between cannabis use and eye disease in this population, it may be prudent for individuals with thyrotoxicosis to avoid using cannabis or to consult with their physicians before using it. As medical marijuana is used to treat a variety of medical conditions, including some that may overlap with thyrotoxicosis, it is important for physicians to be aware of this potential risk and to consider alternative treatment options if necessary. Additionally, patients using medical marijuana should be counseled about the potential risks and benefits of cannabis use, including the potential risk of developing orbitopathy, and advised to use it only under the guidance of a healthcare professional.

Despite the prevalence of dermopathy being greater among cannabis users in the study cohort, we did not discover a significant association between cannabis use and thyroid dermopathy. Previous research has connected tobacco smoking to virtually all extrathyroidal manifestations of thyrotoxicosis, including thyroid dermopathy. However, there is little evidence to suggest a similar association between cannabis use and dermopathy. Our study identified several other independent predictors of thyroid dermopathy, including race, existing orbitopathy, and income level. These findings suggest that socioeconomic factors may play a role in the development of thyroid dermopathy. The study also found that admission to hospitals in the southern region and Asian or Pacific Islander descent were associated with lower odds of dermopathy, although the reasons for these associations are unclear. Finally, our study did not find a significant association between cannabis use and length of hospital stay in patients with thyrotoxicosis.

The current study had some limitations that need to be acknowledged. Firstly, the NIS database used in this study lacked detailed information regarding the severity or duration of thyrotoxicosis, as well as the time interval between diagnosis and the development of orbitopathy or dermopathy. Another limitation was the possibility of bias associated with retrospective studies, such as the use of predefined data. Furthermore, the NIS records hospitalizations for thyrotoxicosis rather than individual patients, which raises concerns about the possibility of duplicate entries of the same patient. Lastly, the use of NIS is subject to coding norms, which increases the likelihood of coding errors. However, despite these limitations, this study made use of substantial sample size, precise coding, and comprehensive analysis, which provide a deeper understanding of the research topic.

## Conclusions

The index study sheds light on the role of cannabis use in thyroid-associated orbitopathy development, which has significant implications for thyrotoxicosis patients. The findings highlight the necessity for further research on the impact of cannabis on eye disease and the underlying mechanisms. As cannabis use increases globally for both medical and recreational purposes, it is crucial to comprehensively understand its effects on health outcomes, including thyroid function and eye health. This study provides critical insights into the risks and benefits of cannabis use for thyrotoxicosis patients, representing an essential step in this direction.
